# Online interventions to help college students to improve the degree of integration of their argumentative synthesis

**DOI:** 10.1007/s11145-021-10248-0

**Published:** 2022-01-11

**Authors:** María Luna, Ruth Villalón, Isabel Martínez-Álvarez, Mar Mateos

**Affiliations:** 1 Department of Psychology and Health. Faculty of Health Sciences and Education, Madrid Open University (UDIMA), Crta. De la Coruña Km. 38,500, vía de servicio número 15, Collado Villalba, 28400 Madrid, Spain; 2 Department of Education, Faculty of Education, Universidad de Cantabria (Spain), University of Cantabria (UC), Avenida Los Castros, 5, 39005 Santander, Spain; 3 Department of Education. Faculty of Health Sciences and Education, Madrid Open University (UDIMA), Crta. De la Coruña Km. 38,500, vía de servicio número 15, Collado Villalba, 28400 Madrid, Spain; 4 Department of Basic Psychology, Faculty of Psychology, Autonomous University of Madrid (UAM), Ivan Pavlov, 6, 28049 Madrid, Spain

**Keywords:** Synthesis writing, Argumentation, Academic writing, Online learning, Critical questions

## Abstract

Writing an argumentative synthesis is a common but demanding task, consequently undergraduates require some instruction. The objective of this study was to test the effectiveness of two interventions on integrative argumentation: one of them was focused on the product features of argumentative texts; and the other one on the processes involved in the written argumentation. Sixty-six undergraduate students participated voluntarily. As an academic task, they were asked to write a pre-test synthesis after reading two sources which presented contradictory positions about an educational issue, then to read two new texts about a different but equivalent issue, and write a post-test synthesis following one of two types of instructional virtual environments. The instructions, implemented in Moodle, presented similar tools, employing videos, graphic organizers, and exercises. The first condition (n = 33) focused on the linguistic features while the second (n = 33), focused on the process, including explicit instruction and a script with critical questions to guide the reading and writing processes. In this study we have also analyzed how the students in the process condition answered some of the critical questions. The results show that the level of integration of the written products improved in both conditions, although this improvement was more pronounced in the process intervention. Nonetheless, the products that achieved medium and maximum integration were still limited. Despite the lack of a relationship between how students answered the critical questions and the level of integration in their post-test, the case analysis highlights certain educational implications and further research.

**Supplementary Information:**

The online version contains supplementary material available at 10.1007/s11145-021-10248-0.

## Argumentative synthesis from multiple sources

Writing an argumentation from sources can be a powerful task for learning (Del Longo & Cisotto, 2014; Mateos et al., 2018; Nussbaum, 2012). However, it is also a demanding task for secondary school and university students (Hyytinen et al., 2016; Lehmann, 2020; Vandermeulen, et al., 2020; Wingate, 2012).

The ability to integrate is essential for synthesis writing. According to Barzilai et al. (2018), integration refers to “*connecting*, *combining*, *or organizing information from different texts to achieve diverse aims such as meaning-making, problem solving, or creating new texts''* (p. 4). Integration from multiple texts is a difficult endeavor even when dealing with complementary sources (Luo & Kiewra, 2019; Wiley & Voss, 1999). Concerning writing an argumentative synthesis from contradictory texts, integration not only involves intratextual and intertextual integration (Segev-Miller, 2007), but also a consideration of the contradictory positions conveyed in the sources to write a text which reflects one's own position regarding the controversy.

Thus, a key element of argumentative synthesis writing is the ability to critically evaluate and integrate arguments and counter-arguments. It involves understanding, evaluating, weighing, combining and generating arguments and counter-arguments from different sources and perspectives to support a final position (Anmarkrud et al., 2014; Mateos et al., 2018). Following Nussbaum (2008) this can be done by writing a persuasive or a reflective text. The former promotes the development of a position supporting it with arguments in favor and evidence. By contrast, reflective writing further encourages the analysis and integration of different positions to produce a reasoned written conclusion. The strategies that can be implemented when integrating arguments from different positions are varied: refutation, weighing and synthesis (Nussbaum & Edwards, 2011). The three integration strategies are challenging, even for college students (Kobayashi, 2015; List et al., 2020; Nussbaum, 2012). Refutation implies considering positions different from the one defended but it seeks to discredit them (showing one-side reasoning), whilst in the process of weighing and synthesizing the advantages and disadvantages of the different positions are recognized, weighted and ranked, in order to find a compromise solution between them. Therefore, writing an argumentation by weighing and synthesizing to reach a new integrative solution promotes higher critical thinking and deeper learning (Mateos et al., 2018).

## Teaching argumentative writing

In spite of its difficulty and complexity, argumentative writing is scarcely taught (Castelló, et al., 2012; Solé et al., 2005), therefore, students require instruction to improve their synthesis skills (Ferretti & Lewis, 2013). To do so, teaching may have to different approaches: one focusing on how written products should be -the most common perspective-, or one with a focus on guiding the processes that writers should display when composing those products (González-Lamas et al., 2016; Mateos et al., 2018). From both approaches, that is, “product” or “process”, a range of aids can be provided. Regarding specifically the integration of multiple sources, teaching usually contains explicit instruction, graphic organizers, practice, etc., in order to support the selecting, organizing, and connecting processes that students need to carry out (Barzilai et al., 2018). Rijlaarsdam et al. (2017) proposed certain design principles that can be used to develop interventions, so instructions should foster building an appropriate task representation, activating prior knowledge, providing sufficient opportunities to practice and encouraging a metacognitive reflection on writing. Within this framework, Van Ockenburg, Van Weijen and Rijlaarsdam (2019), have defined learning activities as organized, cognitive or metacognitive activities, which promote the achievement of learning. Therefore, the activities proposed to the students are mediating variables between instruction and learning outcomes.

Several studies point out how learning activities can be successful in secondary and higher education to foster argumentative synthesis. For example, González-Lamas et al. (2016) designed and applied two programs for students aged 14–16 years old. Both interventions included the same kind of learning activities with an explicit instruction of the different types of arguments and counter-arguments, a collaborative practice, modeling, and finally, the teacher's feedback on their essays. Although the learning/teaching activities were similar in both interventions, the content taught and the abilities demanded were different. One of the two interventions, closer to traditional teaching, was focused on knowing and identifying the linguistic and formal characteristics of the written product of an argumentative synthesis. Conversely, the other intervention was focused on knowing and practising the processes to follow during the elaboration of an argumentative synthesis, guiding them with a script that served as support during the collaborative practice. Both interventions were effective, and all participants made progress in the arguments selection and organization of the arguments. However, only those who received the latter intervention, focused on the process, were able to improve their integration of arguments and counter-arguments from two sources in their products. As far as we know, this kind of comparison has not been undertaken in higher education.

Mateos et al. (2018) evaluated the effectiveness of two types of interventions, both with a process-approach, aimed at improving the argumentative writing of a synthesis of contradictory information on the same controversial topic from different sources. In this study in higher education, both interventions provided a graphic guide with questions, combined with collaborative practice, to help students to be aware of the processes involved in writing an argumentative synthesis. However, in one intervention, additionally, the participants received explicit instruction on the processes for selecting, contrasting and integrating arguments through explanations and modelling of these processes involved in writing argumentative synthesis. Only the group of students who received this second intervention improved their ability to identify arguments and counter-arguments from the conflicting sources and to integrate them by weighing both positions or even offering an alternative viewpoint beyond them.

In line with these two previous studies, in the present one we want to continue contrasting the effectiveness of different instructions, more focused on teaching about the products or about the processes, and using different aids.

Several studies have shown that aids such as guides or exercises may work as tools that can help to enhance the argumentation process. Thus, Mateos et al. (2018) affirm that the guide, in combination with explicit teaching and collaborative practice, can be considered as a scaffold that regulates the argumentation process. Despite the existence of research employing instructional scaffolds, only a few studies have addressed how students actually used them during the instruction. For example, List and colleagues (Du & List, 2020; List et al., 2020) analyzed the students’ notes, and graphic representations, etc. that the participants carried out to show their integration process while integrating multiple sources. Nussbaum and Putney (2020) performed research on what they called *intermediate learning points*, analyzing student’s notes, answers to critical questions, and discourse in an undergraduate course. Through a case study, Nussbaum and Putney were able to observe how, over time, a student learned to establish general principles and to integrate arguments and counter-arguments to reach an integrative solution. The *intermediate learning points* reflected that the strategies worked on during the course resulted in a more innovative and in-depth written product. Taking this into account, in the present study we pay also attention to how the participants use the aids offered to them in the process intervention.

## Online interventions to foster argumentative writing

Open universities and the availability of virtual campuses at traditional or on-site universities have been increasing in the last decade. Moreover, writing interventions in online environments have increased their importance in education in recent years, and even more so due to the current crisis of COVID-19 (Limpo et al., 2020). Virtual training sessions may have a number of advantages, but they are not a simple face-to-face translation (Luna et al., 2020). Furthermore, two elements are usually more important than in face-to-face environments. On the one hand, time is usually a very scarce resource for distance university students, so it is important to know if a brief intervention could make an impact on their abilities. On the other hand, since they do not have the direct motivational help of the instructor, it is even more relevant than in face-to-face environments that students feel that the training is worthy and viable and feel satisfied with the intervention provided.

Some studies have used technologies to implement virtual scaffolds for writing with successful results (Limpo et al., 2020; Strobl et al., 2019). In a recent study, Luna et al. (2020) carried out a first intervention in an online environment, to help students to write integrative syntheses from conflicting sources. The instructional setting included explicit instruction, modeling and practice exercises on the features of the argumentative synthesis products, as elements that have been proven as helpful instructional components (Butler & Britt, 2011; De la Paz & Felton, 2010; Ferretti & Lewis, 2013; Wingate, 2012). This short training was implemented in Moodle and compared to a control group that had only practice. It was successful at improving, on the one hand, undergraduate students’ adjustment to a canonical structure for argumentative texts (including an introduction, body and conclusion) and, on the other hand, the degree of integration between the arguments of the sources achieved by the students. As previously explained, in terms of viability, it is important that students feel satisfied with the instruction they have received and perceive that it is a worthwhile use of their time and effort. In this case, Luna et al. (2020) asked their participants to rate their overall satisfaction and they found that it was high. However, there was still room for improvement in the degree of integration because not many of the participants were able to integrate weighing or synthesizing. For this reason, in this context it is relevant to continue exploring the effectiveness of other proven aids displayed in an intervention focused on teaching about processes that could also be well valued by students.

To sum up, our research team has carried out several studies employing different instructional components with the common goal of helping students to integrate conflicting perspectives. In secondary education we compared an intervention focused on the products with another that focused on the processes (González-Lamas et al., 2016). Later, we tested different instructional components aimed at supporting the processes involved in writing an argumentative synthesis with undergraduate students (Mateos et al., 2018). More recently, we implemented an intervention focused on the features of the argumentative written product with university students in a virtual environment (Luna et al., 2020).

## The present study

In the present study, we have continued contrasting interventions aimed at improving students' degree of integration in distance higher education. We compare two interventions based on the same instructional design principles and presenting the same phases (following Rijlaarsdam et al., 2017) but they differed in some learning activities and tasks, which led to different specific orientations of the instructions (product vs. process). In this case, we have explored whether an intervention which provides learning activities based on explicit instruction, building a graphic organizer, and practice, focused on the synthesis process, can be successfully implemented in this context, and prove to be more effective than that based on the features of the argumentative written products (Luna et al., 2020).

In both cases the task required undergraduate students to construct a written argumentative text from information provided by two divergent sources on a controversial topic in education. The two interventions were aimed at helping students to systematically consider both the arguments in favor and against, the two different positions and the importance of connecting arguments from the two texts to reach an integrated conclusion about the controversy. All the resources and activities that constituted the training were implemented in the Moodle platform using the ‘quiz’ resource. Following the same design principles, the two interventions promote the representation of the synthesis task and meaning attribution, activate and connect knowledge through meaningful learning, foster learning by modeling and promote the active practice of students and self-regulation. The phases that students had to follow to develop their learning process were: problem centered, activation of prior knowledge, explanation of new information and the application and integration of new knowledge. Within the phases, the instructions and tasks that the students performed were different in the two interventions. The ‘product intervention’ dealt with the linguistic and formal elements of argumentative texts with a video lesson comprising a modeling part on how to analyse the text structure and arguments in a written product. Furthermore, the instruction provides written exercises to learn the characteristics of argumentative texts, to identify types of argumentation, arguments, counter-arguments and argumentation fallacies, and to use linguistic elements appropriately, e.g. textual organizers, connectors. The instruction makes students aware of the structure of well written argumentative texts and of the importance of integrating the opposing positions. By contrast, the ‘process intervention’ provided the students with a video lesson containing explicit instruction and modeling on the processes followed by models on integrating, and a written virtual guide. The guide was developed based on diagrams and critical questions that had been used in a prior study (Mateos et al., 2018), presenting an instructional package aimed at supporting the reading and writing processes involved in an argumentative synthesis. The function of these questions was to try to help students identify and weigh the importance of each argument and counter-argument, contrast the two positions, reach an integrative solution, organize ideas to write the conclusion, and revise their final text. One of the sections specifically helps students to integrate the conflicting perspectives posed by the different text sources by weighing or synthesizing them, which is particularly relevant to this study.

The two interventions also differed in the way of following the instruction: the product-training provided the instruction first, and when the students had finished the training they were asked to write their synthesis, therefore it was more similar to how it is usually taught in the classrooms. In contrast, the students using the process-intervention were asked to write the final text as they were working in the instructional environment—consistent with process-focused teaching. Table 1 below describes the instructions and tasks, highlighting the differences between the product and the process intervention.

**Table 1:** Differences between product vs. process intervention within each common phase

Phase	Type of intervention	
	Product	Process
Problem centered: Representing the aim of the instruction by reading a short text	The students of both interventions begin two different Moodle quizzes. Both have an initial section which briefly introduced the whole instructional setting, indicating that the objective of the training was:	
	To get to know better argumentative texts	To improve their skills to read and write argumentative texts
Activation of existing knowledge and demonstration and the acquisition of new knowledge: Explorative thinking fostered by explicit instruction with modeling parts	Continuing from the initial section, the Moodle quiz presented a master class with PowerPoint support and modeling parts inserted. which was recorded in a TV studio. We recommended that the students took notes or opened it in a different window, so that they could watch it again during the training The length and content of the video was different in each intervention:	
	The features of argumentative texts: A 15 min video including, in order of appearance, explanations about the characteristics and objectives of argumentative texts vs. expository texts; linguistic features (opinion verbs, textual organizer etc.). Modeling on how to identify the introduction, arguments, counter-arguments and the conclusion of an argumentative text. An explanation about the types of arguments, the most common mistakes in argumentation and the types of argumentation, i.e. positive, refutation or integrative, which is highlighted as the one that is of particular interest. Instructions about the need to write a text identifying and weighing the arguments of both sources and to establish a conclusion that takes into account what has been said in the texts	The processes involved in reading and writing argumentative texts: A 29 min video including, in order of appearance, explanations about different types of strategies used to write an argumentative text, i.e. (persuasive vs. integrative). Explanations by the lecturer and modeling by two students who are reading the pre-test sources and writing an argumentative synthesis using a guide. They think out loud while they identify the arguments, explore positions and contrast them through a graphic organizer, draw an integrative conclusion, write and revise the final text. The lecturer explains the guide that they are using showing on the screen the meaning of the different critical questions included in it. Finally, instructions are given about the next steps: to give their previous opinion on the topic, download two new texts, and write an integrative argumentation using the aids presented through the next sections of the Moodle quiz
Applying new knowledge Integrating new knowledge	Analyzing and generating texts containing the features of argumentative texts: Next sections of the quiz presented the following exercises: Exercise 1: The students were asked to identify the expository fragments of an argumentative text and copy-paste them in the space for the answer Written feedback was provided immediately after sending the answer Exercise 2: A new short text was presented. It lacked a title, paragraph divisions, textual organizers and connectors. The students were asked to copy it in the space for the answer and to improve it by introducing the missing elements Exercise 3: The next question briefly explained that it is was crucial to identify and create different types of arguments. It then asked the student to build a short text providing arguments about a topic, including underlining different types of arguments, and at least one false argument that they have to create A link with further information about the different types of argument was provided in case they wanted to explore this topic further	Evaluating and integrating both sides of an issue through a diagram before developing a final conclusion on a controversial topic: The next section of the quiz presented a 10 min video tutorial which explained how to use the Padlet tool to create the graphic organizer, as a prewriting tool based on V diagrams (Nussbaum, 2008) to foster argument-counter-argument integration The students build their graphics, including in different columns and rows, the arguments, their evidence and the value attributed to them It is was suggested that the students could finish their organizer by placing in the first rows the arguments that they valued as most relevant
	Analyzing the self- learning process: The next question provided an 11-min tutorial video. This was aimed at teaching students how to create diagrams with Padlet and, specifically, one that makes explicit their process of knowledge acquisition. The students were asked to create a Padlet showing their previous knowledge about argumentative texts, their new knowledge, and their doubts Finally, instructions were given about the next steps: to give their previous opinion on the topic, download two new texts, and write an integrative argumentation on their own	Reading and answering critical questions based on Nussbaum & Edwards (2011) to contrast and integrate arguments, and to write and revise a final text that students had to write simultaneously: From this point on, critical questions in the quiz were presented on each page / section with a space for the students to write their answer. Each section included different critical questions to foster: Section 5: Contrast Section 6: Contrast (continuation) Section 7: Integration: Integrative solution Section 8: Writing Section 9: Writing and revision In addition, Sect. 8 introduced a three-minutes video about how to integrate weighing and synthesizing the arguments from both positions

## Objectives

To sum up, the main objective of this study was to contrast the effectiveness of the two interventions to help undergraduates in a distance university to write argumentative texts after reading sources with controversial positions. One of the interventions focused on the products, specifically on the linguistic, formal, and logical characteristics of the argumentative texts. This intervention has already proven to be useful to improve the level of integration of the students, although in a limited way. Therefore, the other intervention was aimed at training the processes to follow when writing this kind of product, rather than on the products themselves. In addition, in this study we were especially interested in analyzing how the students who used this latter intervention had dealt with the critical questions that fostered the integration processes. Thus, we have presented quantitative and qualitative data to expand our knowledge about how students used these questions.

This piece of research addressed the following hypotheses:

- The two interventions were evidence-based, so both are expected to lead to an increase in the degree of integration.
- Nevertheless, the process intervention included explicit instruction about the processes contrasting the arguments and integrating them via weighing and/or synthesizing the positions which were developed through explanations, a guide with a graphic organizer, and critical questions to scaffold those processes. As these are important integrative argumentative strategies (e.g., Mateos et al., 2018; Nussbaum, 2012), it is expected that the level of integration will be improved to a greater extent in the process group.
- A positive relationship is expected between the approach in which students answer the questions that promote integration in the process-intervention, and the level of integration achieved in their final synthesis texts.
- Finally, it is expected that students will be satisfied with the process-intervention, because the training provides a balance between offering a set of key scaffolds and demands a relatively short amount of time.

## Method

### Participants

Sixty-six undergraduate students, who were attending their first or second year in the Degrees of Education and Psychology in a distance university in Spain, volunteered to participate and completed all the task’s steps (Age = 33.85 years-old, *SD* = 7.92; 90% female). They belonged to a class group of 93 students. 81.7% of the students started the activity and 78.5% completed it. 90.4% of the students who started the activity agreed to collaborate with the study.

The intervention was offered as a class activity while they were taking the subject ‘Psychology of Learning’. The students were informed that the quality of their products would not be taken into account for their grade in the assignment, but this depended rather on a later written reflection about the learning process itself. This writing does not form part of the data used in this study. The students were randomly assigned to either the product-intervention (*n* = 33) or to the process intervention (*n* = 33) group. The ethical requirements of the University were fulfilled and every student was offered the possibility of having the materials for the other intervention once the activity had been completed. All the participants were native speakers of Spanish and regular users of the Moodle platform because it is the primary online learning platform used in the performance of their learning activities in every subject of their degree.

### Materials

#### Source texts

We employed two pairs of source texts on educational topics, in the field of assessment and evaluation that presented conflicting views. Each pair of texts contained the same number of arguments for each perspective, i.e. nine for the pre-test and five and six for the post-test text pairs. All the texts were equivalent in the number of words (between 630 and 815) and readability (Szigriszt-Pazos index^1^ between 44.8 and 56.8).

#### Virtual guide

The students in the process condition had to use a guide, adapted from a previous study (Mateos et al., 2018), with an instructional package aimed at supporting students to write syntheses from two sources including conflicting information. Following Nusbaum (2008), a question format organized into sections was used. The first of these sections asked and taught the students to create a graphic tool. Its aim was to identify and list the controversial topic, the arguments and the counter-arguments of the opposing positions put forward in the two texts, and to write their evidence and relevance down. They were also given specifically reserved spaces to answer the next questions. As shown in Table 1, the critical questions helped the students to weigh the importance of the arguments and counter-arguments, to compare and contrast the positions, to select and organize ideas for the conclusion, and to revise their final draft. In this paper we are going to focus on Sect. 7, which deals with the integration process. The questions included were the following: *Does any single position carry greater weight?, Why?, Is there any means of reconciling two positions?, Is there any new alternative position that will integrate the different positions?, Is there a position which only holds if certain conditions occur?* (The Screenshots in Appendices 1–3 illustrate what the students’ saw before accessing the Moodle quiz, part of the virtual guide and its Sect. 7).

### Procedure

This study presents an experimental pre-post design in which we compare two interventions. The independent variables were the condition (product/process). The dependent variables were the degree of integration in the synthesis texts written by the students, and students’ level of satisfaction with the training.

Within the academic task, the students in both conditions were asked to: (1) fill in a questionnaire to gather initial student’s data; (2) write a synthesis after reading two sources which presented contradictory positions, about an educational issue as a pre-test; (3) read two new texts about a different but equivalent issue and, following one of two types of instructional virtual environments; (4) write a new synthesis as a post-test (see Table 2).

**Table 2:** Sequence, elements, and technological tools of the two interventions

PRE	Intervention		Post
	Linguistic	Process	
(1) Sociodemografic data and ethic consent (2) Synthesis 1	Video lesson (Introduction + Modeling on how to identify arguments and text structure)	Video lesson (Introduction + Modeling on how to read, identify arguments, contrast and integrate positions and write the final text)	(1) Synthesis 2 (2) Students’ assessment of the intervention
	Lesson: exposition + exercises on argumentative texts features	Graphic organizer to contrast the arguments presented in the sources	
	Graphic organizer to think about what students knew on argumentation	Lesson: exposition + questions on the writing processes of an argumentative synthesis	
	Clouser	Clouser	

The participants were told to try to start and finish the task whenever they wanted but to complete it in only one session, in order to collect real data about the time spent carrying out the assignment. Most of the students spent about two hours (between one hour and fifteen minutes, and three hours and forty-five minutes) in the product-intervention and about four hours (between one hour and a half, and six hours and twenty minutes) in the process intervention (remember that students who received the process intervention wrote their synthesis post-test while following the instruction).

Regarding the students' evaluation of the intervention, after the intervention they were asked to report, on a 1–10 scale, to what extent they were satisfied with the intervention.

Specifically, in this study we have focused on the analysis of the integration process. For this we have carried out an analysis of one section in the Moodle quiz, Sect. 7, which presented different critical questions focused on the process of reflecting on the possibility of reconciling the two positions presented, reaching an integrating conclusion.

### Scoring

Each synthesis was coded regarding its degree of integration following Mateos et al. (2018), on a 0 to 6 scale (see Table 3). The first three levels (0, 1 and 2) showed one-sided reasoning; the third takes into account the two sources but just to refute one of them, while those syntheses that scored level four and above showed integration via weighing and/or synthesising.

**Table 3:** Levels of overall integration in the synthesis

Overall argumentative strategy	Type and frequency of specific argumentative strategy	Presence and type of final conclusion
0. Personal opinion, not based on source texts	Gives their personal opinion on the topic without including arguments from the texts. The argument is based on an argument from the texts but wanders far from them	
1. Neutral	Does not take a clear position. Describes or lists arguments for both positions, either in a linear way, laying out all arguments for one position and then all the arguments for the other, or by alternating arguments for both positions	Does not conclude, or concludes by asserting that both positions must be taken into account, i.e. the pros and cons of both positions, without integrating them in any way. Therefore, they are neither weighed nor synthesized into a new position
2. Argues in support	Takes one of the two positions and argues in favour of that position without considering the opposing position. May include some refutation or integration but basically relies on the defended position Lists arguments for both positions and concludes by taking a stance in support of one of the positions	Does not conclude, or concludes in support of one of the two positions Concludes by taking a stance in favour of one of the two positions
3. Integration via refutation	Takes a position in support of one of the two perspectives and argues in its favour while refuting the opposing perspective. Approximately 50% of arguments used are rebuttals	May not have a conclusion. However, if there is one, it may be in favour of the supported position and/or in opposition to the refuted position
4. Minimum integration via weighing or synthesizing	Takes a position in support of one or both perspectives and argues by weighing or synthesizing arguments from both positions, i.e. two integrations, throughout the text Includes two integrations, one in the body of the text and another in the conclusion Includes two integrations, both in the conclusion	Does not contain an explicit conclusion or contains a conclusion that is not integrative, this can be neutral or in support of one of the positions Contains a partial conclusion Contains a conclusion with two integrations
5. Average integration via weighing or synthesizing	Takes a position in support of one or both perspectives and argues by weighing or synthesizing arguments from both positions, i.e. integrating at least two throughout the text Weighs or synthesizes arguments from both positions throughout the text, i.e. integrating three or more	Contains a conclusion that is partially integrated, which may have been used earlier in the text or be new Does not contain an explicit conclusion, or the conclusion is neutral
6. Maximum integration via weighing or synthesizing	Integrates throughout the text, at least twice Presents both positions	Contains a conclusion that is an overall integration Contains a conclusion that weighs or synthesizes various arguments, i.e. more than two on each side

The first author coded each synthesis. The second author coded 48% of the sample. The kappa value for the inter-rater agreement was 0.82 and disagreements were settled by discussion.

Additionally, for the process condition, we carried out an analysis of the participants’ answers in the section of the guide that included the critical questions that especially promoted the integration of perspectives (Sect. 7). Table 4 shows that we considered to what extent they had adjusted to the demand proposed by the questions, and the degree of integrating the two positions that they showed when answering them. In the critical questions that promoted the integration of positions, the first three levels do not reflect an authentic answer to the questions posed. Thus, either they did not allude to arguments, or they took a position using arguments from the other position but only to refute them. On the contrary, from score 3, they did reflect considering the opposite perspective: they took a position explaining the need to take into consideration at least one argument from the other perspective, as an important condition for the solution. Finally, with score 4, participants showed a preliminary integration, taking into account various conditions.

**Table 4:** Levels of use of Sect. 7: Reconciliation of positions

Score/criterion	Definition	Samples from students’ answers
0. No answer	No answer is offered, or only responds with either ‘yes’ or ‘no’ without including information from the source texts	No, because the two positions have their advantages and disadvantages (LMS)
1. General stance	A specific position is taken and explained in a general way without taking into account the arguments in the texts	To me, the position that has the most weight is that education has to create autonomous and competent people … In order to establish an objective school performance evaluation system and to start operating, the Spanish government should invest in creating a random and unknown system of evaluation (IU)
2.Claim that one position is more grounded than the other one	Takes one of the two positions and argues in favor of that position without considering the opposing position. It may include some mention of the other position but without explanation. Basically, relies on the defended position	I think that the position in favor has more weight because it refers to studies carried out previously, which exempts it from subjectivity and generalization … I firmly believe that both positions express their ideas very well, although the position in favor always tries to find feasible solutions and relies on ‘positive’ arguments and not so much ‘disqualifying’, as the position against does (IM)
3. Stance including just one condition of the opposite perspective	Takes a position in support of one of the two perspectives and argues in its favor, whenever an argument from the other position is overcome, as a condition	The approach of the different positions would be possible as long as the evaluation was diversified (more subjects) in a less standardized way, and this could be adjusted to the diversity of educational systems (countries) … I think that position 2 would accept the external evaluation if the results will be applied to introduce the measures that produce an improvement in the educational system and a deeper evaluation of the different educational systems will be carried out (FT)
4. Preliminary integration	Refers to a tentative but preliminary solution on the issue by considering at least one argument from one position and two arguments, as conditions, of the other perspective	Currently the discussion is open, and no way has been found to reconcile the two positions, but it is right that a conciliatory act could be attempted by evaluating the variables that are considered in the external evaluations, as well as the comparatives that are performed between different countries. The totality of subjects that comprise the curriculum should be taken into account, as well as the factors that have an influence on the quality of learning. Another point that could be highlighted is what is valued and for what? By this I mean that any element of the educational process is worthy of being evaluated, the curriculum, the center, the teachers or their training (JR)

The first and the third authors acted as judges and evaluated all the students’ answers. The inter-judge agreement was achieved by means of Cohens’ Kappa and was 0.90. Where agreement was not reached, a third judge, who was another researcher with similar expertise, decided the final coding.

Regarding the students’ evaluation, we asked the participants to grade their overall satisfaction on a 1–10 scale.

## Results

In order to test the hypotheses, we carried out several analyses. Firstly, we obtained descriptive results for all the variables and tested the assumptions. Since the homoscedasticity of variance was fulfilled, we continued with an F-test. Thus, secondly, to ascertain the effectiveness of both interventions on the degree of integration, we performed a repeated-measures ANOVA. The between-subject factor was the condition (product/process) and the within-subject variable was time (pre/post). Thirdly, we carried out an analysis of correlation to test if there was any relationship between the students' answers to the critical questions included in Sect. 7 of the virtual guide and the post-test degree of integration. Finally, we conducted a cluster and a qualitative analysis on the students' answers.

### Degree of integration

**Fig. 1:**
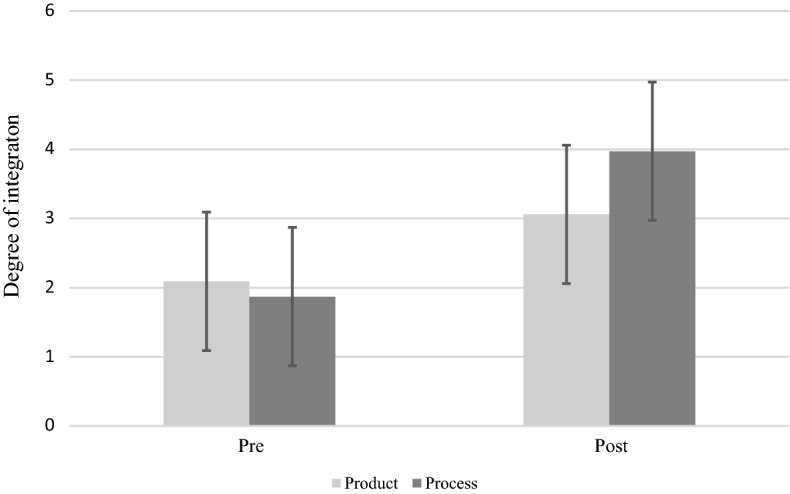
Mean and confidence level (95%) for degree of integration by time and condition

As can be seen in Fig. 1, before the intervention, the students' synthesis average value in both groups showed no integration, with score 2 being the most frequently occurring value. By contrast, after the training, the mode reached a value of 2 in the product-group and of 4 (minimum integration) in the process-group.

The ANOVA test indicated an interaction between time and group [(*F* (1,66) = 5.15, *MSe* = 2.01 *p* = 0.027, *ŋ*^*2*^_*p*_ = 0.074)]. Additionally, a main effect of time was found, meaning that both groups performed better in the post-test (*F* = 38.38 *p* < 0.001, *ŋ*^*2*^_*p*_ = 0.38). The Bonferroni post hoc test also revealed significant differences according to time, showing that the scores of the students in the two groups improved over time (*p* < 0.001). In addition, the results did not show significant differences between the conditions in the pre-test (*p* = 0.23). Thus, the participants started with the same level of degree of integration and both groups increased after the instruction, but to a greater extent those in the process condition.

The results indicate that both groups increased their scores after the training, reaching, on average, the minimum level of integration only in the process-condition.

### Students’ evaluation of the intervention

We performed a descriptive analysis to address to what extent the participants in both instructions were satisfied with the intervention. Only some of the students reported this data. Regarding their evaluation of the training, the results showed that students in the process-intervention were overall satisfied (*n* = 26, *M* = 7.6, *ST* = 1.17).

### Use of the virtual guide

We coded the students' answers in Sect. 7 of the process intervention into the categories as explained in Table 4. The distribution of students in each category is shown in Table 5.

**Table 5:** Distribution of participants in each category per section in Sect. 7

Category	Number of participants
0	3
1	5
2	3
3	7
4	15

The results indicate that more than a half of the participants (22 out of 33) gave responses categorized at score 3 or higher. However, only 15 students scored 4, achieving a preliminary level of integration.

We performed Spearman correlations between the integration in the post-test and the scores in Sect. 7 (see Table 6) but no significant relationships were found.

**Table 6:** Summary of correlations between integration in the post-test and the scores in Sect. 7

Measure	1	2	3
1. Pre-integration	–	.15	.23
2. Post-integration	.15	–	.26
3. Section 7	.23	.26	–

^*^p < .01

Therefore, there is no linear relationship between the answers to the integration questions in the guide and the integration level shown in the final text written by the participants. In order to understand this lack of relationship more profoundly, we decided to explore the students’ patterns based on their previous level of integration, their scores in Sect. 7, and their subsequent level of integration in the posttest.

The main axis of our analysis was the final level of integration reached by the students, considering also their scores in the pre-test. In a second step, we considered their answers to Sect. 7. Considering the first axis, we established two groups of students: a large group (n = 24) who maintained or improved their level of integration in their products, and a smaller one who did not show integration either in their initial or their final texts (n = 9). Among these two main groups, consistent with the lack of correlation, we found a variety of students’ behaviours both in the products and in their answers to Sect. 7. Taking a closer look at this variety, we found that in the first group four students started from syntheses that already showed integration and preserved or improved that level of integration in their post-test synthesis. The other 20 participants started from non-integrative syntheses and came to elaborate final texts with at least a minimum level of integration (score 4 or above). Conversely, the nine participants in the second group did not achieve a score above 3 in any of their synthesis texts.

As can be seen in Table 7, of the participants in the first group, i.e. the 24 who developed integrating final products, we found 13 who gave answers with scores of 4 in Sect. 7. These students are the ones who showed the most expected behavior based on our hypotheses: they maintained or improved their level of integration in their products and made their integrating ideas explicit in Sect. 7 of the guide. Another three participants in this group also approximate this profile: they reached level 3 in Sect. 7 showing that they had at least taken into account the integration of one argument from each text. The cases that showed behavior which differed to that expected in this group were the eight students who, despite maintaining or advancing in their level of integration from their pre to post- tests, gave answers of 0, 1 or 2 in Sect. 7, in other words, they did not explicitly show in the guide how the two positions could be integrated. Despite this, those participants did express an integrative conclusion in their final texts. These cases illustrate the situation of students who were able to take advantage of the questions in the guide, although they did not make it explicit in the designated space the integrations that they later included in their final text.

**Table 7:** Summary of students’ integration patterns over the intervention

GROUP 1 (n = 24):			GROUP 2 (n = 9):	
Participants who maintained or improved their level of integration in their final products			Participants who did not show integration neither in their initial nor in their final texts	
Maintained or improved their level of integration in the final products and made their integrating ideas explicit in Sect. 7 (n = 13)	Integrative final texts but just a minimum level of integration in Sect. 7 (n = 3)	Integrative final texts but no explicitation on how the two positions could be integrated in Sect. 7 (n = 8)	Neither integrative final texts nor explicit integration in their responses in Sect. 7 (n = 4)	No-integrative final text but at least minimum level of integration in Sect. 7 (n = 5)

The second group of participants is made up of the nine students who started from developing non-integrative texts and who did not improve after the intervention. We expected that these individuals would not show integration by their responses to the questions in Sect. 7. This was clearly the case in four of the students, who obtained scores of 0, 1, or 2. However, the other five participants gave answers in Sect. 7 which were scored at level 3 or 4. Especially striking was the case of two students who mentioned several arguments that could be taken into account to reach a compromise solution in their answers to the guide (level 4 in Sect. 7) but who did not write integrative texts: they basically just argued in favor of one of the positions. In these cases, we observed in their answers to the guide, that they had clearly thought and reflected on possible conditions to reach an integrating solution of the two positions. However, when they wrote their final texts they did not textualize those elements.

## Discussion

This paper presents an experimental pre-post design in which we compare two interventions implemented in Moodle aimed at fostering undergraduates’ synthesis writing from contradictory sources. Both interventions were based on the same design principles, presented the same phases and included equivalent technological tools but employed some different learning activities and tasks. In the product-intervention the environment presented an instruction focused on the argumentation product features, dealing with linguistic and formal aspects of argumentative writing, and types of arguments and argumentation fallacies. In the process intervention the instruction focused on the reading and writing processes of writing an argumentative synthesis. Our hypotheses expected a greater improvement in the degree of integration in the process-intervention, a positive relationship between the use of the guide and the final integration achieved, and a positive evaluation of the training by the students.

The results show that synthesis texts written by the students in both conditions were similar in the pre-test but, after the intervention, the level of integration of the written products was higher in both conditions, although more in the process intervention. These results concur with previous research (Ferreti & Lewis, 2013; González-Lamas et al., 2016; Mateos et al., 2018), because explicit instruction on processes with the help of a written guide as a scaffold is more useful than other types of instructional aids. This work reaffirms that an intervention focused on the writing process can be more effective in improving integration than another focused on the products’ features. In addition, although the process intervention presented here was shorter than face-to-face teaching, we now have evidence that this type of training could be successful in a distance-learning context. The fact that the participants were satisfied with the intervention is another explanation that reinforces this idea.

Moreover, in this study we have presented a case analysis on how the students in the process intervention actually used the scaffold provided in the instruction. Specifically, the analysis carried out on the students’ answers to the guide, aimed at promoting the integration of positions, in line with what suggested by Nussbaum and Putney (2020), enabled us to access intermediate points in the students’ learning progressions. Contrary to what was expected, we did not find any correlation between the level of integration that students showed in the section of the guide that encouraged this process, and the level of integration in their final products. This might be due to the different ways that the students used the guide and their learning progression. In fact, we found two groups of participants: a large group of students comprised of those who wrote integrative final products, and a smaller one of those students who did not. However, there were several patterns among both of the groups. Thus, as was expected, in the former, we found a high number of participants who showed integration in the critical questions and maintained, or improved, their level of integration in the final product. Therefore, it would appear that the guide was useful for most of the students. In the latter group, there were students who did not write integrative final texts. These students appeared not to have taken advantage of the guide (neither of the whole training), since they did not show integration, neither in their answers to the questions nor in their written products.

Nonetheless, in both groups there are striking cases. On the one hand, in the group that managed to integrate there were students who did not answer the guide at all. However, despite this, they wrote a more integrative product in the post-test. In this case the questions might have acted as a prompt to foster the integrative process although they did not write anything. This was also observed in the research by Luo and Kiewra (2019), who reported that the students did not always write on all the materials provided.

On the other hand, in the group that did not manage to integrate, there are two students who wrote non-integrative synthesis in the pre-test, achieved the maximum score in integration in the critical questions, but still did not write an integrative pos-test product. Thus, we observe in their answers to the guide that they had thought and reflected, at least in a schematic way, about two or more possible conditions to reach an integrating solution of the two positions. However, when they wrote their final texts, they did not show any integration. This might either be due to their lack of ability to textualize, or to an inappropriate task representation, which still led them to produce a one-sided reasoning. Their final texts were elaborated arguing in favor of a position, mentioning the arguments in both texts, but without explaining the solution that may integrate them.

Nevertheless, an important conclusion is that none of the students who showed integration in the critical questions got a lower score in the post-test than in their pre-test. In fact, in most cases it seems that the questions had helped them to write better synthesis than in the pre-test.

To conclude, as a result of the qualitative approach presented here, we acknowledge that there is not a simple straightforward relationship between the pre-test, the use of the guide, and post-test; students may have employed the scaffolds in very different ways and some of their answers (or lack of them) to the guide may not reflect their cognitive processes. However, the training helped most of the students to improve their level of integration.

In spite of the general improvement in the integration in our sample, the products that achieved medium and, especially maximum integration, were still limited. These findings suggest that, even with explicit instruction on the process, students struggled to reach high integrative conclusions and that they still needed more support to develop their skills for generating integrative conclusions. Integration is a very demanding process, much more than selecting and organizing, as students in the Luo and Kiewra (2019) study acknowledged. One possible explanation for this limited success is that the students needed more prior knowledge about the subject, since this is an important requirement when arguing (Lehmann, 2020). Another possible explanation, which concurs with the findings of Luo and Kiewra (2019) is that some students, even in higher education, still have difficulties in textualizing, that is, writing down their texts in real time. Precisely, the last result of our case analysis points in this direction. Perhaps, if the students in our process condition had received more specific aids on textualizing they would have been able to reflect and expand the seeds of the integration that they had previously shown during the training provided. A third possible explanation may be linked to the brevity of the intervention. A more intense practice might help students to obtain a greater improvement in their synthesis integration scores.

However, by asking the students to answer the questions in the specific spaces reserved we were able to obtain indirect data on students’ task representation while using the virtual guide, and to speculate on the problems some may have had in writing their argumentative synthesis. This information could lead to the design of more suitable scaffolds.

### Limitations and further research

We only had a small sample and we found a diversity of students’ answers to the critical questions. This particularly affected the quantitative analysis. Future studies should have a larger number of participants to elucidate to what extent the results found here are more or less common among undergraduates.

With respect to other studies in this field of research, on this occasion we did not address the differential effectiveness of each of the instructional components, for example explicit instruction or written guide alone, but instead we implemented complete packages that included various scaffolds. Therefore, we can compare the effectiveness of an instruction focused on the processes to another focused on the products, but we cannot know the effectiveness of each type of aid included in them. Further studies could investigate the impact of the different instructional components in virtual environments. Furthermore, they could also take into account students’ variables regarding their learning profiles, which may enrich our knowledge about the most appropriate aids for each of them (Gil et al., 2010; González-Lamas et al., 2016; Vandermeulen, De Maeyer, et al., 2020). Understanding the suitability of different scaffolds could be a great step in personalizing student learning through online environments.

Furthermore, we have used an ecological tool and setting, although we did not monitor whether the students were using other resources, for example pen and paper for taking notes, instead of using the guide or complementing it. Moodle does not enable data gathering on this issue, but future research could employ Inputlog, or video recording, for example which could provide more information on the students’ process (Du & List, 2020; List et al., 2020; Vandermeulen, Van den Broek et al., 2020). This information could be useful, for example, for a deeper understanding of the students’ processes, particularly in the cases of those who may have used the guide as a prompt without writing in it.

Finally, regarding the students’ satisfaction with the intervention, this issue should be addressed more qualitatively, because it remains unclear why the participants were satisfied. Our work did not ask for reasons or explanations and therefore we do not know if the students valued the training for example because they generally enjoyed the intervention, or because they thought the materials were useful, or for some other reasons. Likewise, we have not enquired about what aspects the students did not appreciate. Moreover, it is worthy to remember that these findings are based on self-reported data, since we did not access students’ actual behaviour but rather their thoughts on how the aids helped them.

Despite these limitations, this study illustrates how technology has been used to implement a successful intervention on argumentative writing in an online education context and sheds more light on how students deal with a virtual guide.

## Supplementary Information

Below is the link to the electronic supplementary material.

Supplementary file1 (DOC 406 kb)

## Footnotes

1 Szigriszt-Pazos is the current reference for the Spanish language readability. A text with a score of 50 to 65 is considered to be average.
