# Supplementary material for: Online interventions to help college students to improve the degree of integration of their argumentative synthesis
Source: Read Writ. 2022 Jan 11;36(4):937–63. doi: 10.1007/s11145-021-10248-0 (PMC8749917; doi:10.1007/s11145-021-10248-0)

Appendix A

A1. View of the interventions in the virtual classroom in Moodle


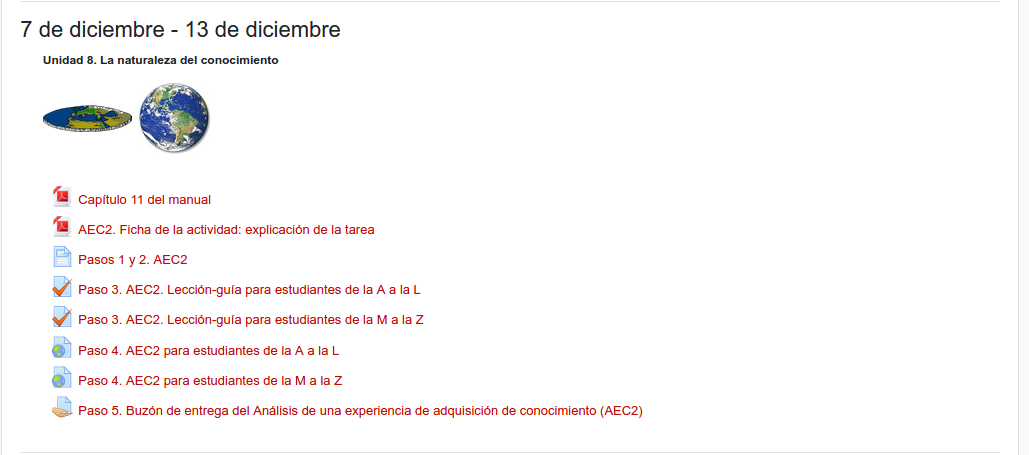


A2. View of the Section 1 of the Moodle quiz: the video-lesson


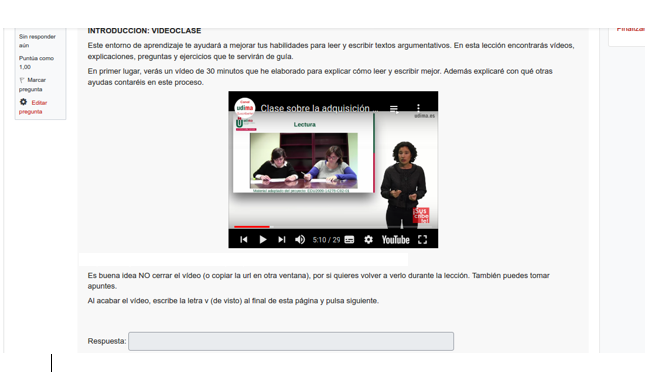


A3. View of the Section 4 of the Moodle quiz: the graphic organizer


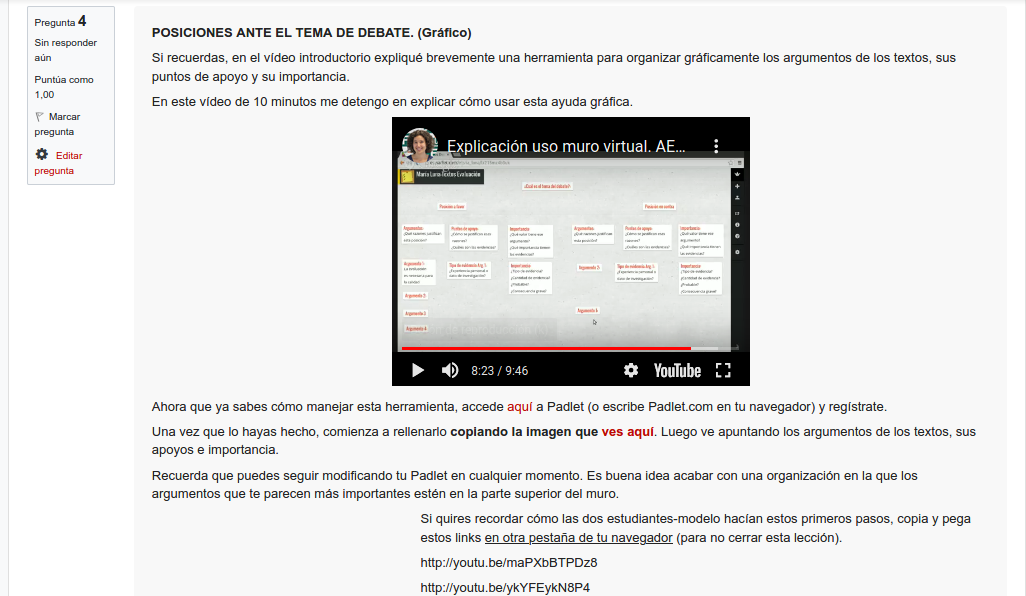


A4. View of Section 7 of the Moodle quiz: the critical questions to foster integration and a fragment of a student’s answer


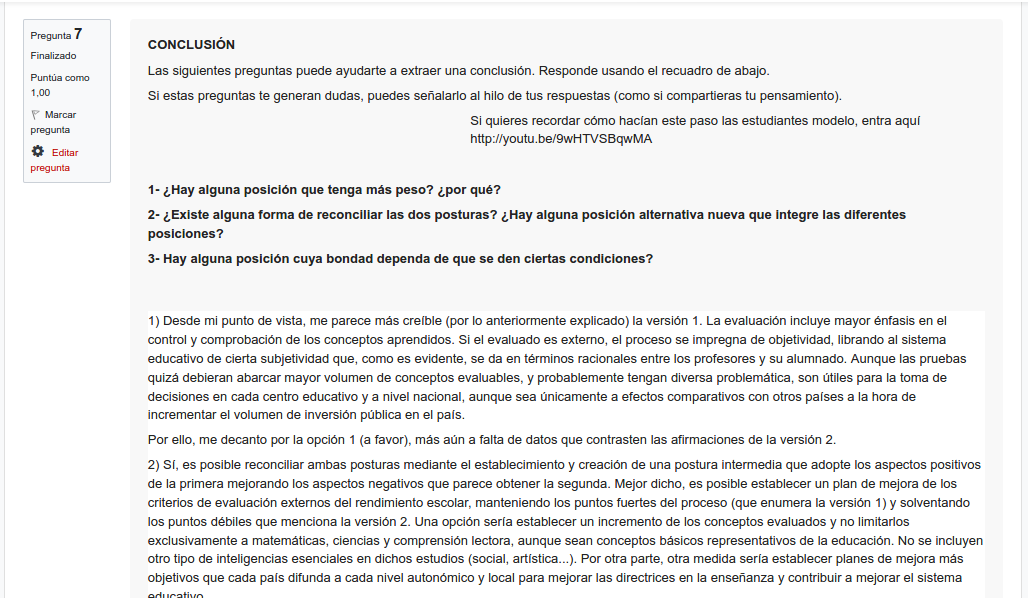

Supplement: Supplementary file 1 — Supplementary file1 (DOC 406 kb) [file 11145_2021_10248_MOESM1_ESM.doc]
